# Ashes to Rashes: An Exploration of the Intersection Between Smoking and Cutaneous Lupus Erythematosus

**DOI:** 10.3390/biom15091250

**Published:** 2025-08-29

**Authors:** Rafael O. Homer, Ahmed Eldaboush, Darae Kang, Nada S. Ahmed, Touraj Khosravi-Hafshejani, Ming-Lin Liu, Victoria P. Werth

**Affiliations:** 1 Corporal Michael J. Crescenz VAMC, Philadelphia, PA 19104, USA; rafael.homer@pennmedicine.upenn.edu (R.O.H.); daraek@sas.upenn.edu (D.K.); nada.sayed@outlook.com (N.S.A.); lium1@pennmedicine.upenn.edu (M.-L.L.); 2 Department of Dermatology, Perelman School of Medicine, University of Pennsylvania, Philadelphia, PA 19104, USA; 3 Clinician Investigator Program, Faculty of Medicine, University of British Columbia, Vancouver, BC V5Z 3X7, Canada

**Keywords:** cutaneous lupus erythematosus, smoking, tobacco, skin, immunopathogenesis, oxidative stress, autoimmunity, neutrophil extracellular traps, extracellular vesicles, epigenetics, inflammation, type I IFN

## Abstract

Cutaneous lupus erythematosus is an autoimmune skin disorder with a known association with cigarette smoking. Smokers with cutaneous lupus have a worse disease course and may be refractory to treatments. Despite many studies documenting this association, minimal work exists examining the molecular drivers of these clinical differences. This review delves into how cigarette smoke may influence key immunopathogenic pathways in cutaneous lupus, including oxidative stress, interferon signaling, inflammatory cell recruitment, extracellular vesicles, and immune regulation. Additionally, factors such as epigenetics and heat injury are considered as well. Here, we integrate the existing and emerging literature on the pathophysiology of cutaneous lupus with known effects of cigarette smoke on the skin and immune system and propose hypotheses that may explain clinical differences in smokers. Understanding the molecular underpinnings of these differences may yield a clearer picture of the disease and more effective treatment strategies.

## 1. Introduction

Cutaneous lupus erythematosus (CLE) is an autoimmune skin disease with a well-documented association with smoking [1,2,3,4,5,6,7]. A high prevalence of smoking has been reported across various CLE subtypes, with most studies being conducted in higher-income countries [4,5,6,8,9]. The described smoking rates amongst patients with CLE are reported to be higher than the national prevalence in the corresponding general populations [4,5,6,8,9]. Globally, there are 1.3 billion tobacco users, with 80% living in low- and middle-income countries [10]. Given this large number of smokers, secondhand smoke (SHS), which includes exhaled smoke and smoke directly from lit tobacco products, represents an important route of exposure for nonsmokers [11]. SHS has numerous effects on the immune system and is linked to a range of health issues [11,12,13,14]. In addition, third-hand smoke (THS), defined as the residual cigarette smoke (CS) that accumulates on indoor surfaces and objects, and in dust, can interact with the skin, penetrate the barrier, and affect multiple types of cells in the dermis and epidermis [15,16]. With the pervasiveness of smoking, it is important to consider it as an environmental factor even in patients with CLE who are not active smokers.

CLE is recognized as an interferon-driven autoimmune skin disease that involves both innate and adaptive immune mechanisms [17,18]. This skin condition encompasses a spectrum of subtypes, including acute cutaneous lupus erythematosus (ACLE), subacute cutaneous lupus erythematosus (SCLE), and chronic cutaneous lupus, of which discoid lupus erythematosus (DLE) is the most common subtype [17]. CLE can occur independently or as a manifestation of systemic lupus erythematosus (SLE) [17]. Lesions will typically appear in sun-exposed areas and are characterized by erythema, scale, and variable degrees of scarring or atrophy [17].

CS has been repeatedly associated with the onset, persistence, and exacerbation of various autoimmune conditions, including cutaneous manifestations of SLE, with cumulative pack-years being linked to greater cutaneous damage [19,20,21]. Smoking has been associated with the diagnosis of DLE, as well as with higher disease severity and a resistance to antimalarial therapy in DLE [4,5,9,22,23,24,25]. In SCLE, smoking is similarly linked to increased disease severity and poor treatment response [4,9,24]. Associations between smoking and lupus erythematosus tumidus and poor response to antimalarials have also been demonstrated [9,23]. For ACLE, data from SLE patients show smoking is associated with a higher likelihood of active malar rash [19,20]. Furthermore, several studies show that in general, CLE remission is negatively associated with smoking [26,27,28]. CS has been shown to be phototoxic, causing significant dose-dependent and UV-dependent photohemolysis, which may counteract the photoprotective effects of antimalarial medications commonly used in CLE [29,30,31]. Recent work also suggests that smoking is associated with decreased long-term treatment cessation of mycophenolate mofetil and methotrexate in patients with CLE, suggesting that even second or third-line treatment options for patients refractory to antimalarials are impacted by CS [32]. While smoking is known to alter drug-metabolizing enzymes like CYP1A2 and CYP2E1 [33,34,35], this does not appear to explain treatment resistance in CLE. Studies demonstrated that this mechanism did not account for the reduced efficacy of antimalarials, pointing instead to other interactions [36]. Collectively, these findings establish smoking as a key obstacle to achieving effective therapeutic control in patients with CLE.

Specific interactions between CS and CLE remain underexplored. CS is known to have direct effects on the skin, impairing wound healing, accelerating cutaneous aging, and increasing susceptibility to infection [37]. Proposed mechanisms for CS-induced autoimmunity are varied, encompassing autoantibody generation, epigenetic alterations, enhanced proinflammatory cytokine release, compromise of tissue barriers, and its role in genetically susceptible individuals [38]. In CLE specifically, it has been proposed that the disease is exacerbated through a variety of mechanisms that are discussed in depth in this review. These mechanisms include increased inflammatory cytokines, oxidative stress, apoptosis, extracellular vesicles (EVs), and DNA damage [8,24,39].

Two important reviews have shaped the foundation for this work. A 2016 review offers an extensive exploration of how CS affects both adaptive and innate immune responses, emphasizing molecular mechanisms that may be pertinent to autoimmunity [40]. The mechanisms that underlie leukocytic differences in smokers continue to be explored [41,42,43,44], and research on the effects of CS on specific immune cell subsets is ongoing [45]. A 2022 review presents a thorough synthesis of CLE immunopathogenesis, focusing particularly on Type I interferon-mediated inflammation, and cellular-level and molecular aspects of the disease [46]. Although these reviews are foundational, neither directly investigates how smoking influences immune dysfunction in CLE, a research gap the current review endeavors to bridge.

**Figure 1:**
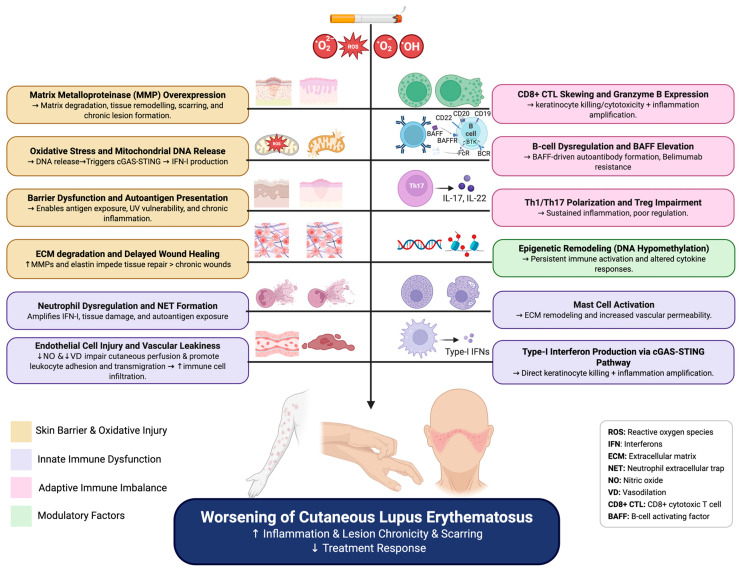
Summary of proposed mechanisms driving worsened disease course in smokers with CLE Created in BioRender. Eldaboush, A. (2025) https://BioRender.com/guju204 (24 July 2025).

This review synthesizes evidence on how cigarette smoke influences key immunopathogenic pathways in CLE, including interferon signaling, oxidative stress, inflammatory cell recruitment, and immune modulation (Figure 1). To build upon the existing literature, we performed a literature search using PubMed and utilized work undertaken by our group and others to propose mechanistic hypotheses to explain the variable impact of CS on immune regulation and inflammation in CLE. The literature on smoking’s immunologic impact is complex, and at times contradictory, likely reflecting variability in studies and differences in experimental hypotheses and conditions. However, what is clear is that cigarette smoke profoundly influences immune homeostasis, and understanding its role in CLE could inform both treatment decisions and disease prevention strategies.

## 2. Initial Insults: Cigarette Smoke and Damage Induction in CLE Skin

### 2.1. Oxidative Stress and DNA Release

Oxidative stress and DNA damage are key contributors to cutaneous inflammation and may serve as an important mechanistic bridge between CS exposure and CLE pathogenesis. Cigarette smoke is a potent inducer of oxidative stress in skin cells, including both keratinocytes and fibroblasts.

In human keratinocytes, CS exposure leads to the loss of scavenger receptor class B type I (SR-B1), a process mediated by hydrogen peroxide derived from CS gas and cellular NADPH oxidase activity [47]. Other studies have shown that CS causes significant oxidative damage to keratinocytes [48,49]. This oxidative insult may disrupt normal keratinocyte function, survival, and, ultimately, barrier integrity (Table 1).

Fibroblasts are similarly vulnerable. In vitro studies demonstrated that cigarette smoke extract (CSE) exposure leads to a dose- and time-dependent decline in fibroblast proliferation, accompanied by features of cellular senescence and increased oxidative stress [50]. These effects are thought to result from the depletion of antioxidant enzymes such as superoxide dismutase and glutathione peroxidase [50]. Additional studies examining tertiary smoke have shown concentration-dependent cytotoxicity in murine fibroblasts and nicotine-driven vacuolization in human fibroblasts [15]. Importantly, both oxidative stress and mitochondrial DNA release are recognized activators of the cGAS/STING pathway, leading to Type I interferon production [51,52]. Given the centrality of IFN-I in CLE, smoking-induced activation of this pathway via oxidative stress in keratinocytes and fibroblasts may represent a key mechanism linking environmental exposure to disease activity (Table 1).

Finally, CS has been shown to impact endothelial cells (ECs) through the delivery of exogenous reactive oxygen species (ROS) and induction of endogenous ROS production (via NADPH/xanthine oxidase), leading to endothelial damage, reduced nitric oxide bioavailability, and impaired vasodilation [53]. These changes may impair cutaneous perfusion and promote leukocyte adhesion and transmigration, contributing to CLE lesion formation (Table 1).

**Table 1:** Known cellular effects of cigarette smoking on non-immune cells located in the skin.

Cell Type	Key Effects of Smoking/CS Exposure	Potential Impact on CLE Pathogenesis	Citation(s)
Keratinocytes	Induces oxidative stress, mitochondrial DNA release, and increased MMP-1 expression.	May trigger the cGAS-STING pathway to produce Type I IFN; degrades the extracellular matrix.	[16,47,48,49]
Fibroblasts	Causes oxidative stress, cellular senescence, and upregulation of MMP-1 in dermal fibroblasts.	May contribute to matrix remodeling and fibrosis seen in DLE; may participate in the inflammatory feedback loop.	[15,50,54]
Endothelial Cells	Increases ROS, reduces nitric oxide, enhances leukocyte adhesion and permeability.	May impair cutaneous blood flow and promotes the infiltration of inflammatory immune cells into the skin, fueling lesion formation.	[53,55,56]

### 2.2. Formation of Extracellular Traps and Extracellular Vesicles

A key aspect of how smoking may interact with CLE involves the recruitment and activation of neutrophils. In smokers with CLE, an increased presence of neutrophils in lesional skin was observed and associated with worsened disease activity [57]. CS not only increases peripheral neutrophil counts [58,59,60] but also modifies neutrophil behavior by inhibiting chemotaxis and increasing neutrophil extracellular trap (NET) release [61]. In fact, CS-induced NETs were found to activate plasmacytoid dendritic cells (pDCs), suggesting a role in immune crosstalk [62].

NET formation results from the extracellular release of chromatin by neutrophils following nuclear envelope and plasma membrane rupture [63]. Recent studies from our and other groups have revealed that NETotic nuclear envelope rupture is triggered by PKCα-mediated phosphorylation of lamin B [64] and CDK4/6-mediated phosphorylation of lamin A/C [65], which is subsequently followed by the breakdown of the plasma membrane involving the cytoskeleton [66]. The disruption of these membranes allows for the externalization of nuclear components such as DNA, histones, and other nuclear autoantigens. Therefore, neutrophils may contribute to inflammatory responses through the formation of NETs, which can propagate autoimmunity, and further release can be intensified by CS exposure. NETs are extracellular lattices of DNA (mitochondrial and nuclear), histones, matrix metalloproteinases (MMPs), neutrophil elastase, myeloperoxidase, and various other components extruded by activated neutrophils as part of the innate immune response [63,67,68]. The release of these components liberates a diverse array of damage-associated molecular patterns (DAMPs), which can function as endogenous danger signals that trigger cascades of aberrant immune activation [63,68].

This mechanism is highly relevant to smokers. Oxidative stress is a known trigger of NET formation [63,68,69], and smoke-induced oxidative environments may create a self-perpetuating cycle of ROS production and subsequent NET release in CLE lesions. Furthermore, the smoker-specific metabolite, thiocyanate, has been shown to directly stimulate NET release [61]. The clinical importance of this pathway is highlighted by observations that NETs are abundant in the lesions of certain CLE subtypes, including lupus panniculitis, acute cutaneous SLE, and DLE, suggesting its importance in lesional inflammation [70].

In addition to NETs, tobacco smoke stimulates the release of EVs from immune and other cells. Smoking has been shown to induce proteolytic EVs from macrophages and can trigger immunomodulatory or immune-associated EVs [39,71,72]. Notably, in neutrophils, tobacco smoke was shown to induce proteolytically active ADAM17-positive EVs [73]. The latter finding is particularly significant given that recent work has demonstrated that inactivation of a protective ADAM-17 lupus pathway exacerbates photosensitivity [74]. Thus, in smokers with CLE, neutrophils may introduce a destructive and unregulated source of EV-derived active ADAM-17 that is distinct from the protective pathways. Recent work also demonstrates that keratinocytes release EVs that help mediate immune-crosstalk in UVB-induced skin inflammation [75]. One untested hypothesis is that CS may induce immunomodulatory EV release from keratinocytes in CLE.

While the literature examining CLE-specific EVs is minimal, research in related conditions is promising [76]. In similar disorders such as SLE and dermatomyositis, EVs are being explored for their role in pathogenesis and as potential diagnostic or therapeutic targets [77,78,79]. Collectively, smoke-induced EV release and NET formation may amplify local inflammation and barrier damage, with both being potential areas of future exploration.

### 2.3. Barrier Dysfunction and Autoantigen Presentation

Barrier dysfunction is a feature of CLE pathology, particularly in DLE, where lesions may heal with atrophic scarring [80]. Similarly to the microvascular injury induced by lupus-associated vasculitis and UV [81], smoking may also exacerbate CLE through its direct effects on the microvascular bed of the skin. Smokers have been shown to exhibit diminished endothelial-dependent vasodilation and reduced post-ischemic hyperemia in forearm microvessels [55]. These findings imply that chronic smoking impairs cutaneous blood flow and endothelial reactivity, potentially altering skin barrier integrity, oxygenation, and inflammatory surveillance, which are crucial in CLE pathogenesis (Table 1).

Recent work has demonstrated that dermal fibroblasts from SLE patients exhibit a heightened inflammatory cytokine response, mirroring the inflammatory profiles observed in CLE lesion biopsies [82]. Importantly, one study identified TGF-β as an upstream regulator of fibrosis in DLE [82]. While scarring lesions exhibited collagen upregulation, non-scarring lesions were characterized by more prominent inflammatory pathway activation, underscoring the dual roles of fibroblasts in immune signaling and matrix remodeling [82].

In addition to fibrosis, barrier degradation in CLE lesions may be driven by matrix MMPs, particularly those expressed in epidermal keratinocytes. MMPs have been shown to be upregulated in CLE lesions and correlate with disease severity [83]. Their expression is further enhanced by UV radiation, which compounds tissue damage in photosensitive patients [83,84,85]. CS is also a known inducer of MMP expression in both skin and vascular tissues, contributing to collagen degradation and premature skin aging [54,86,87,88]. TSE has also been shown to trigger macrophage release of EVs containing bioactive MMP-14 [39], which has gelatinolytic and collagenolytic activities and regulates collagen homeostasis in adult skin [89]. This implies a role for infiltrated monocyte/macrophages in tissue damage and collagen remodeling in CLE smokers. Nicotine, a key component of CS, has been shown to upregulate COL1A, elastin, and MMP-1 in dermal fibroblasts, along with markers of apoptosis and cell cycle disruption [54] (Table 1). In vitro studies on HaCat keratinocytes exposed to THS demonstrated increased MMP-1 expression, an exaggerated proinflammatory response, and delayed wound healing, reinforcing the impact of tobacco products on barrier integrity [16] (Table 1). CLE lesions contain increased mast cells, with distribution varying by subtype [85,90]. Mast cells may further contribute to barrier disruption and matrix remodeling. Tobacco-derived materials have been shown to activate mast cells in vitro and increase the production of mast cell-secreted proteinases, which can degrade extracellular matrix components [91,92].

Elevated MMP activity in CLE smokers may impede tissue repair and further perpetuate inflammation. Some studies suggest that MMPs have a protective effect through the degradation of circulating immune complexes in SLE [93]. In smokers with CLE, the potential dual role of MMPs as immune complex modulators and drivers of tissue damage poses interesting questions about their net effect on tissue damage. Clarification on how smoking-induced MMP activity intersects with CLE-specific pathways may uncover new therapeutic or preventative targets and help understand the role of MMPs in the disease.

In CLE, molecules such as NETs and damaged cells may lead to autoantigen generation and downstream immune sensitization to self. Smoking may also independently generate autoantigens. As studied in other autoimmune contexts like rheumatoid arthritis, tobacco smoke was found to associate with protein citrullination and vimentin carbamylation, leading to smoking-dependent autoantigen generation [94,95]. While this mechanism is well-described in rheumatoid arthritis, a direct link between smoking, protein citrullination, and a specific autoantibody profile in CLE has not yet been established and remains an area for potential investigation. In general, SLE has mixed evidence regarding smoking’s association with autoantibody production. A 2005 study found that smoking was associated with a higher risk of dsDNA seropositivity compared to never smokers [96]. However, a 2014 study found no clear association between smoking status and individual autoantibodies in SLE patients, unaffected first-degree relatives, or healthy controls, but did find that current smoking was associated with positivity for at least one autoantibody (excluding ANA) in SLE subjects [97].

More recent prospective studies using the Nurses’ Health Study cohorts have provided further insight into smoking and specific SLE subtypes. One study found a strong association between current smoking and anti-dsDNA-positive SLE, which also extends to other specific SLE subtypes [98]. The association between smoking and CLE-specific autoantigen generation remains to be studied and may present a different story from that seen in rheumatoid arthritis and SLE.

## 3. Amplification of Inflammation: The Interplay of Innate and Adaptive Immunity

### 3.1. Inflammatory Cell Recruitment and Activation

CLE lesions are defined by sustained inflammatory cell infiltration, with evidence suggesting that CS may amplify this process by modulating both stromal signaling and immune cell activation.

CS has been shown to stimulate NF-κB activation in ECs, which enhances leukocyte adhesion and transmigration [56]. Moreover, dysfunctional ECs exhibit increased permeability, allowing the passage of immune cells into tissues and causing inflammation [56]. This suggests that smoking-induced vascular leakiness may promote autoantigen exposure and amplify immune infiltration in CLE (Table 1).

CS may intensify immune infiltration by inducing proinflammatory cytokine production. CS and nicotine have been shown to stimulate human macrophages to release IL-8 [40], a cytokine involved in the recruitment and activation of neutrophils and the promotion of angiogenesis [99]. Additionally, exposure of pDCs to CSE reduces their production of TNF-α, IL-6, and IFN-α in response to TLR9 stimulation, while enhancing IL-8 secretion [100]. Together, these changes may shift the cytokine environment toward neutrophil-predominant inflammation.

Tobacco smoke extract (TSE) has been shown to cause the release of high mobility group box 1 (HMGB1), a nuclear protein that serves as a danger signal that mediates sterile inflammation, both as a soluble protein and in association with EVs from macrophages [101]. Adding another layer of complexity, trends in macrophage phenotypes in smokers and patients with CLE appear to be at odds. Gene expression analysis in SLE and DLE demonstrates a polarization of macrophages toward the proinflammatory phenotype, although DLE lesions also contain a sub-population of macrophages co-expressing M1 and M2 markers, indicating a hybrid or transitional activation state [102,103]. In contrast, CS exposure, as shown in studies on murine and human macrophages, appears to skew macrophages toward an anti-inflammatory M2 phenotype and impairs their phagocytic capacity [40]. This presents a potential paradox in CLE. While CS may promote M2-like anti-inflammatory polarization, the simultaneous increase of IL-8 and neutrophil recruitment in a disease state already marked by neutrophil dysfunction [104] may ultimately overwhelm any anti-inflammatory benefit. It is interesting to note that elevated CD163, an M2 macrophage marker, was shown in the skin and sera of SLE patients [105]. Whether M1 or M2 macrophages dominate in CLE smoker lesions remains unknown, but resolving this question may explain some clinical variability in CLE tobacco users.

### 3.2. Cytotoxic Amplification of Inflammation

Emerging research suggests that smoking may affect CLE through its modulation of cytotoxic CD8+ T cells and granzyme B expression. Granzyme B is of particular interest, as its levels have been shown to be associated with disease activity, kidney damage, and increased interferon signature in SLE [106,107]. A 2020 study using single-cell RNA sequencing and mass cytometry on human peripheral blood samples found that smoking leads to a loss of naïve CD8+ T cells and an expansion in terminally differentiated subsets [108]. Notably, a rare CD16+ CD8+ T-cell sub-population enriched in smokers exhibited natural killer (NK) cell-like properties, including high expression of granzyme B and perforin-1 [108]. A recent in-depth transcriptomic analysis of T cells in CLE found that T cells in the skin had lower levels of activation and cytotoxicity-related genes, including granzyme B [107]. This finding, as supported by immunohistochemistry, suggests that the baseline CLE environment is not characterized by high cytotoxic T lymphocyte (CTL) activity [107]. The observation that smoking may increase granzyme B may suggest a distinct smoking-driven pathological mechanism by which local immune response is shifted toward a more cytotoxic and tissue-damaging phenotype.

In the skin, this signature may be reflected in CLE lesions. One study reported increased endothelial granzyme expression in CLE smokers compared to nonsmokers [57]. Although granzyme B is classically associated with apoptosis, it also exerts extracellular effects that can amplify tissue damage. These include extracellular matrix protein degradation, increased vascular permeability, and induction of angiogenic or injurious remodeling, processes that may contribute to endothelial injury and facilitate immune cell infiltration and sustain inflammation in CLE lesions [109].

Systemically, granzyme B appears to be upregulated in smokers across multiple tissues. Elevated levels have been observed in airway tissue [110], the peripheral blood of active smokers, and in patients with chronic obstructive pulmonary disease (COPD), where aging further amplified levels [111]. However, not all studies agree; one investigation reported no significant differences in peripheral granzyme B expression between smokers, nonsmokers, and emphysematous smokers [112], underscoring the need for tissue-specific analyses.

Nevertheless, the finding of elevated granzyme B in CLE smoker lesions suggests a possible mechanistic role in keratinocyte and endothelial injury. Immunohistochemical analysis of CLE has identified NK cells at the dermal–epidermal junction, colocalizing with CD8+ T cells and pDCs [113]. CTLs are known producers of granzyme B [109], a protein capable of inducing both caspase-dependent and independent apoptosis in target cells [109]. In CLE, granzyme B expression within lesional skin has been shown to correlate with Type I interferon signatures, indicating that CTL-mediated keratinocyte apoptosis may contribute to IFN-driven inflammation [114]. NK cells, although relatively rare in the skin, may contribute to CLE pathology via granzyme-dependent keratinocyte death [113].

CS also appears to modulate pDC frequency and function. Smokers exhibit increased circulating pDCs and total dendritic cells in peripheral blood [115]. pDCs may also participate in this granzyme axis through cytokine crosstalk. IL-21, a cytokine implicated in lupus pathogenesis, can induce granzyme B production in human pDCs, enabling them to promote keratinocyte apoptosis in cooperation with NK cells [113]. Importantly, in CLE lesions, the local balance between IL-21 and IFN-I was shown to regulate granzyme B levels [113]. In murine models, CS exposure increased systemic IL-21 [116], suggesting a possible mechanism by which smoking could modulate pDC-mediated cytotoxicity.

Finally, an early study showed that smoking was associated with an increase in CD4+ T and B lymphocytes expressing Fas, suggesting that these cells may be more susceptible to apoptosis [117]. The increased cytotoxic environment seen in smokers may allow for more autoantigen generation and trigger the cGAS/STING pathway. An examination of cytotoxicity specifically in CLE smokers may be helpful to understand this relationship.

### 3.3. Type I Interferon Cascade: A CenBioRendertral Hub in CLE and Smoking

Core to CLE pathogenesis are Type I interferons (IFNα, IFNβ, and IFNκ), which drive a self-perpetuating cycle involving inflammation, cell death, and immune activation [17]. CS appears to modulate several upstream triggers and downstream amplifiers of this cascade. The precise nature of this modulation is complex and may contribute to both IFN amplification and attenuation.

One study found that cigarette smoke condensate extract led to less Type I IFN responsiveness in mouse embryo fibroblasts, 293T, and HeLa cells through the degradation of the IFNAR1 subunit of the Type I IFN receptor [118]. On the other hand, there are many other ways in which CS may directly or indirectly increase the Type I IFN signal. Indirectly, CS endothelial injury may enhance leukocyte infiltration into the skin, subsequently fueling Type I IFN-driven immune activation, which triggers CLE flares.

CD14+ CD16+ macrophages have been identified as being among the most activated immune cells in CLE lesional skin biopsies, where they were also major producers of Type I interferons IFNα and IFNβ [119]. CS may affect macrophage polarization and function in ways that alter their IFN-producing capacity, as discussed above.

Keratinocytes are recognized as IFN producers, producing both Type I and Type III interferons, along with proinflammatory cytokines and chemokines, in response to various factors, including nucleic acids [120,121]. CS and its byproducts, including THS and nicotine, induce mitochondrial dysfunction and proteomic alterations in keratinocytes, which may further promote cytosolic DNA sensing via cGAS-STING and other interferon pathways [16,122].

Neutrophils may present another link between smoking and IFN-I amplification. As mentioned above, NETs can activate pDCs and contribute to vascular and autoimmune pathology [62,123,124]. In both humans and murine SLE models, NETs, which, as previously stated, include genetic material, have been able to induce IFN-I through TLR9 and the DNA-dependent CGAS/STING pathways, linking neutrophil dysregulation directly to interferon overproduction [125,126].

**Figure 2:**
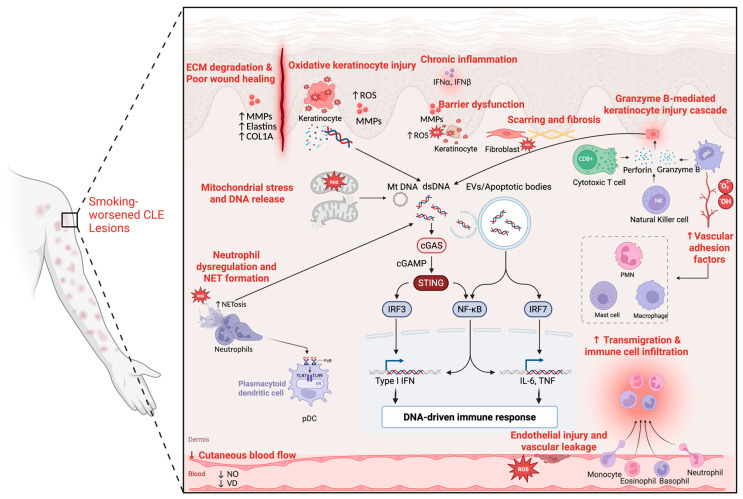
Proposed cigarette smoke-driven skin barrier and innate immune dysfunction in CLE. Created in BioRender. Eldaboush, A. (2025) https://BioRender.com/d5tiaxc (24 July 2025).

Studies on COPD have shown that CS promotes NF-κB-dependent autoimmunity by a mechanism in which DNA from NETs activates the cGAS/TLR9 pathway [127]. COPD studies further demonstrate that CS significantly increases TLR9 expression and cytokine production in pulmonary but not peripheral cytotoxic CD8+ T cells (CTLs) [128]. Nicotine itself may increase TLR9 responsiveness in peripheral cells [129]. While this phenomenon has not been studied in CLE, it raises the possibility that CS-induced TLR upregulation in skin-resident T cells could exacerbate disease or interfere with antimalarial efficacy, which targets TLR7/9 signaling [130,131]. This hints at the dosage inadequacy of antimalarials and aligns with research letters that suggest increasing antimalarial dosage in smokers to overcome increased TLR signaling [28,132]. The interplay of sections two and three is summarized graphically (Figure 2).

## 4. Dysregulation of Adaptive Immunity in Smoking-Related CLE

### 4.1. B-Cell Dysregulation and the B-Cell Activating Factor Axis

B cells are central to the pathogenesis of both SLE and CLE. Loss of B-cell tolerance and aberrant activation contribute to autoantibody production, inflammatory cytokine release, and immune dysregulation [46,133]. In CLE specifically, transcriptional profiling has identified B-cell signatures that distinguish between subtypes, both with and without systemic involvement [134,135].

One key cytokine in B-cell biology is B-cell activating factor (BAFF), which promotes B-cell survival and maturation [136]. BAFF levels are shown to be elevated in the peripheral blood and epidermis of patients with DLE, SCLE, and lupus profundus compared to healthy controls [137,138,139]. BAFF receptor expression has been associated with germinal center maintenance [140], and in murine models, CS enhances BAFF-driven autoantibody responses [141]. Human studies of SLE similarly show elevated serum BAFF in smokers [142].

These findings carry therapeutic implications. Belimumab, a monoclonal antibody targeting BAFF, has demonstrated efficacy in CLE [143]. However, its effectiveness appears reduced in smokers. Studies in SLE show that smokers respond less favorably to belimumab [144], with diminished efficacy in mucocutaneous disease, but not in articular manifestations [145]. Whether smoking similarly compromises belimumab efficacy in CLE remains unknown and warrants further study. Given evidence of BAFF elevation in smokers, it is plausible that CS contributes to autoantibody generation and treatment resistance in CLE through B-cell dysregulation. Finally, current or recent smoking has been linked to elevated levels of the SLE-related cytokine B lymphocyte stimulator (BlyS) and reduced levels of IL-10, further suggesting an interaction between B cells and CS [146].

### 4.2. CD4+ T-Cell Polarization and Regulatory Imbalance

Th1 skewing is particularly prominent in CLE. One study reported an elevated Th1:Th2 chemokine receptor ratio in patients with active CLE [147]. In DLE lesions, IFN-γ-producing Th1 cells dominate the infiltrate [148], a finding further supported by multiple studies [149,150,151]. Murine models have demonstrated that Th1-polarized CD4+ T cells can induce lupus-like skin lesions [152,153], highlighting their pathogenic potential.

Smoking also appears to influence and modulate CD4+ T cells, as peripheral CD4+ T-cell levels are upregulated in smokers [58,154,155,156], and recent in vitro work suggests that NETs induced by CSE may activate monocyte-derived myeloid dendritic cells and promote differentiation of T cells toward proinflammatory phenotypes Th1 and Th17 [157]. In vitro, CSE, in the presence of antigen-presenting cells and activating peptides, enhances Th1-associated cytokine production and upregulates *T*-bet, the master transcription factor for Th1 differentiation [158]. Similar findings are seen in COPD, where patients exhibit elevated Th1 cells and plasma IFN-γ [159]. Additional studies show that CSE, in the presence of IL-12, promotes Th1 skewing via the α7 nicotinic acetylcholine receptor (nAChR) pathway [159]. Given IFN-γ’s ability to induce keratinocyte apoptosis in several skin disorders [160,161], as well as sustaining inflammation, smoking-induced Th1 dominance may act as a driver of lesion chronicity in CLE.

While Th17 cells are also suggested to play a role in CLE, the supporting evidence shows greater variability. Increased Th17 infiltration and serum IL-17 have been observed in DLE and, to a lesser extent, SCLE [149,162]. These findings are supported by murine models (e.g., p-selectin-deficient mice with CLE-like skin and Th17 expansion) [163]. However, a transcriptomic study failed to detect Th17 signatures in DLE lesions [148], underscoring subtype variability and methodological differences. IL-6 plays a key role in Th17 differentiation, especially in the presence of TGF-β [164], and is elevated in CLE skin lesions [165]. Elevated epidermal IL-6 has been reported in DLE and SCLE lesions [165]. A study examining the peripheral blood of lupus patients demonstrated that Type I interferons and Th17 pathways co-regulate pathogenic immune responses in SLE, with IL-6 serving as a key link [166]. Moreover, UVB and TLR stimulation of keratinocytes from lupus patients led to increased IL-6 production, further amplified by Type I interferons [165]. This suggests that the IFN-rich CLE microenvironment may prime skin for Th17 development. Smoking may amplify this process. Smoking has been associated with increased IL-6 levels in both serum [167] and plasma [168], although results vary [169]. Experimental models show that chronic CS exposure increases the Th17/Treg ratio in the peripheral blood of mice [170], and CSE-exposed dendritic cells release extracellular vesicles that promote both Th1 and Th17 polarization [72].

Regulatory T cells (Tregs), which restrain inflammation, may also be impaired in smokers. Smoking may impair regulatory T-cell function by reducing their numbers or promoting the expansion of dysfunctional, non-suppressive subsets [40]. Some models show initial Treg expansion with subacute CS, but reduction during chronic exposure, suggesting dose- and duration-dependent effects [170]. In CLE, Treg deficiency or dysfunction may contribute to disease pathogenesis, particularly in lesional and photosensitive skin, where a lack of immunoregulation may exacerbate inflammation [46,171]. One study found that CLE non-responders to antimalarials had significantly fewer Tregs in lesional skin compared to responders [119]. Since smoking is associated with antimalarial resistance, chronic CS exposure may impair Treg function and exacerbate therapeutic non-responsiveness.

## 5. Epigenetic Implications of Smoking for CLE

The genetic underpinnings of CLE are still being elucidated. Associations have been found with genetic polymorphisms, HLA haplotypes, and complement deficiencies, indicating innate and adaptive immune regulatory pathways in disease susceptibility [172]. Cigarette tar extracts have been shown to cause DNA damage [173]. CS-driven epigenetic modification and genetic damage, especially if immune-related, may contribute to CLE pathogenesis.

Epigenetic mechanisms such as DNA methylation and histone modification are altered in SLE immune cells and tissues, leading to aberrant expression of inflammatory genes [174]. Smoking is a well-established epigenetic modifier, capable of inducing changes that persist long after exposure and influence immune system function [175]. A 2024 study of over 900 healthy adults found that current smokers exhibited DNA hypomethylation at smoking-associated CpG sites, including genes involved in immune regulation and cell signaling [176]. Strikingly, when 11 of these CpG sites were included as covariates in the analysis, the previously observed link between smoking and increased IL-2 and IL-13 secretion after immune stimulation was lost [176]. This suggests that DNA methylation may mediate persistent adaptive immune activation.

Moreover, smoking explained up to 9% of the variation in cytokine responses, similar in magnitude to age, sex, or genetic variation [176]. These findings suggest that smoking may exert long-term or even transgenerational effects on immune regulation via epigenetic remodeling. Enzymes regulating these modifications may be activated by smoking and may drive the expression of inflammatory genes [175]. While this has been studied in SLE and other immune-mediated diseases, epigenetic dysregulation has not been directly explored in CLE, nor has the possibility that CS-induced epigenetic changes contribute to CLE risk, chronicity, or treatment resistance. This represents a gap in the literature and a compelling direction for future research.

## 6. Heat, Nicotine, and E-Cigarettes

The heat generated from tobacco smoke may present another avenue for CLE exacerbation or occurrence. The Koebner phenomenon is a well-recognized occurrence in which inflammatory skin lesions are induced by cutaneous trauma, including friction, blunt trauma, or heat [177]. This phenomenon aligns with “lupus ab igne”, the term proposed for DLE lesions incited by chronic heat exposure from sources like stoves or heating blankets [178]. One case report describes the development of DLE lesions on the lips of a 34-year-old woman with a history of SLE and Sjögren syndrome, who used electronic cigarettes (EC) [179]. The central location of the lesion on her vermillion border and upper cutaneous lip directly corresponded to where the EC mouthpiece rested during use, as confirmed by the patient [179]. Although EC and CS are different, they both may be able to trigger DLE lesions through the Koebner effect.

ECs and both electronic nicotine delivery products (ENDPs) and heated tobacco products are very underexplored areas in terms of their effects on CLE. Minimal epidemiological data examining potential interactions exist, and, to our knowledge, only the above case report touches on CLE associated with the use of ECs. This highlights a significant gap in the literature regarding these nicotine delivery methods. While the specific impact of ENDP-delivered nicotine on CLE pathogenesis requires further investigation, established effects discussed throughout this review suggest that nicotine itself, irrespective of combustion-related toxins, may modify the disease course of CLE. As nicotine-related product usage continues to increase, exploring whether these products cause specific disease modulation is a potential area of future research.

## 7. Conclusions and Future Directions

Smoking is a common environmental factor, both through active tobacco use and through other routes such as SHS and THS, potentially even impacting CLE patients who do not actively smoke. Relatively little research examining the specific molecular intersection of smoking and CLE exists. By integrating the literature on CLE immunopathogenesis with the known effects of smoking, this review highlights a multifaceted process in which cigarette smoke likely initiates and amplifies inflammation through pathways central to CLE. The evidence points toward a model in which smoking contributes to everything from the initial oxidative insult and barrier dysfunction to the dysregulation of B- and T-cell responses. This review has identified potential areas of novel future exploration to better understand the interaction between CLE and CS. Ultimately, counseling and cessation of tobacco usage are likely the best steps forward for patients with CLE who smoke.

Indeed, the evidence strongly suggests that quitting is a direct therapeutic intervention. Studies show that former smokers exhibit significantly lower disease activity [24], and in SLE, former smokers did not have an association with active rashes, whereas current smokers did [20]. Furthermore, quitting may help mitigate the reduced efficacy of first-line antimalarials often seen in active smokers [24]. Second-line immunosuppressants like mycophenolate mofetil and methotrexate may also regain efficacy, with former smokers achieving treatment outcomes comparable to those of never-smokers [32]. This positions smoking cessation as a foundational step in managing the disease and improving therapeutic response.

However, future research into the intersection between CLE and tobacco may help us better understand CLE as a disease and the implications of how CS interacts with drugs. Understanding of these effects and interactions may yield more personalized treatment approaches for CLE patients based on their smoking status if cessation cannot be achieved.

## Data Availability

No new data were created or analyzed in this study. Data sharing does not apply to this article.

## Abbreviations

The following abbreviations are used in this manuscript:

CLE	Cutaneous Lupus Erythematosus
SHS	Secondhand Smoke
THS	Thirdhand Smoke
CS	Cigarette Smoke
ACLE	Acute Cutaneous Lupus Erythematosus
SCLE	Subacute Cutaneous Lupus Erythematosus
DLE	Discoid Lupus Erythematosus
SLE	Systemic Lupus Erythematosus
EV	Extracellular Vesicle
CSE	Cigarette Smoke Extract
EC	Endothelial Cell
ROS	Reactive Oxygen Species
NET	Neutrophil Extracellular Trap
pDC	Plasmacytoid Dendritic Cell
MMP	Matrix Metalloproteinases
TSE	Tobacco Smoke Extract
NK	Natural Killer
IFN	Interferon
COPD	Chronic Obstructive Pulmonary Disease
CTL	Cytotoxic T Lymphocyte
BAFF	B-Cell Activating Factor
Treg	Regulatory T Cell
EC	Electronic Cigarette
ENDP	Electronic Nicotine Delivery Products
